# Supplementary material for: Characterizing cell-type spatial relationships across length scales in spatially resolved omics data
Source: Nat Commun. 2025 Jan 3;16:350. doi: 10.1038/s41467-024-55700-1 (PMC11699133; doi:10.1038/s41467-024-55700-1)
Supplement: Supplementary file 3 — Supplementary Software 1 [file 41467_2024_55700_MOESM3_ESM.zip › CRAWDAD/plots/supplementary/S7_lda_KSFB.predictedBy.PKHL_posterior.pdf]

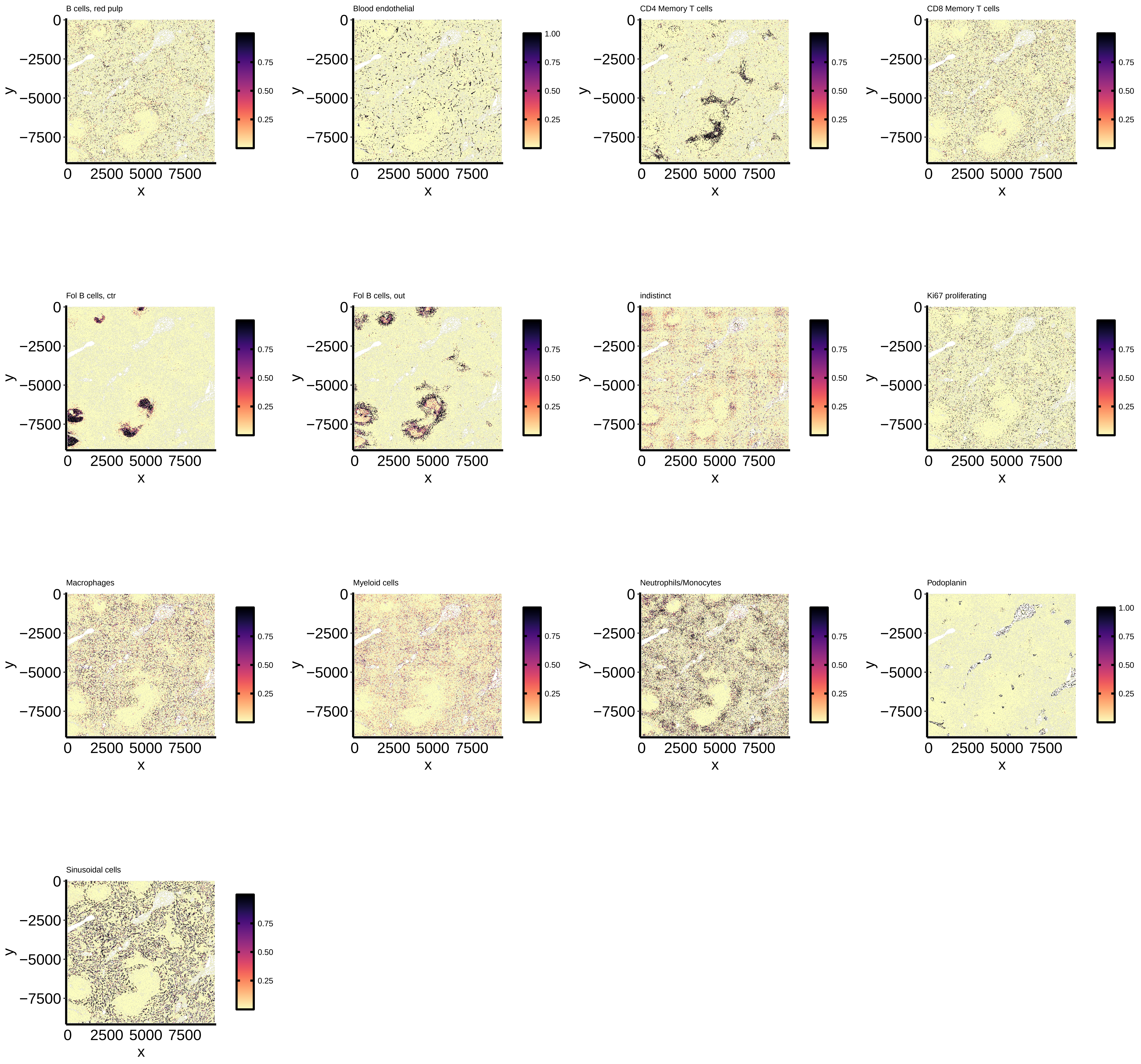
B cells, red pulp
Blood endothelial
CD4 Memory T cells
CD8 Memory T cells
Fol B cells, ctr
Fol B cells, out
indistinct
Ki67 proliferating
Macrophages
Myeloid cells
Neutrophils/Monocytes
Podoplanin
Sinusoidal cells
x
y
0
2500
5000
7500
−2500
−5000
−7500
0.25
0.50
0.75
1.00
